# Cognition and fatigue in clinically stable multiple sclerosis: EDSS and MRI metrics outperform serum biomarkers

**DOI:** 10.1186/s12883-026-04894-6

**Published:** 2026-04-17

**Authors:** Deborah K. Erhart, Luisa T. Balz, Roland Opfer, Lothar Spies, Franziska Bachhuber, Ioannis Vardakas, Daniela Taranu, Stefanie Jung, Tanja Fangerau, Makbule Senel, Kornelia Kreiser, Ingo Uttner, Dorothée Lulé, Hayrettin Tumani

**Affiliations:** 1 Department of Neurology, University Hospital Ulm, Oberer Eselsberg 45, 89081 Ulm, Germany; 2 jung diagnostics GmbH, Röntgenstrasse 24, Hamburg, 22335 Germany; 3 Department of Neuroradiology, University Hospital Ulm, Oberer Eselsberg 45, Ulm, 89081 Germany

**Keywords:** Multiple Sclerosis, PIRA, MRI, BICAMS-M, Fatigue

## Abstract

**Background:**

Fatigue and cognitive impairment are common in multiple sclerosis (MS) affecting up to 95% and 65% of the patients, respectively. Only little is known about the interplay of different volumetric magnetic resonance imaging (MRI) parameters, the serum biomarkers neurofilament light chain (sNfL) and glial fibrillary acidic protein (sGFAP), cognition, and fatigue in MS patients without signs of progression independent of relapse activity (PIRA; i.e. clinically stable). This study aimed to evaluate whether volumetric MRI and serum NfL and GFAP are associated with cognitive impairment and fatigue in clinically stable relapsing-remitting multiple sclerosis (RRMS) and to examine whether the Expanded Disability Status Scale (EDSS) has an added value.

**Methods:**

A retrospective mono-centric cohort of 54 clinically stable (no disability worsening/relapse-free) RRMS patients underwent comprehensive clinical assessment with the EDSS, Brief International Cognitive Assessment for Multiple Sclerosis (BICAMS-M), Fatigue Scale for Motor and Cognitive Functions (FSMC), in addition to established biomarkers of MS pathology, including sNfL, sGFAP, and magnetic resonance imaging (MRI: thalamic/hippocampal/whole brain parenchymal volumes/annualized brain volume loss (BVL/year)). Associations were examined using regressions, correlations, and group comparisons.

**Results:**

Thalamic and hippocampal volumes were significantly lower in cognitively impaired than non-impaired patients (both *p*<.001), while sNfL and sGFAP showed no group differences (*p*>.35; *p*>.09). Fatigue was not associated with MRI or serum markers (*p*>.09). BICAMS-M correlated with EDSS (*r*=–.56; *p*<.001) to a similar extent as with thalamic or hippocampal volumes (*r*=.55; *p*<.001). BVL/year was highly associated with cognitive impairment, explaining 63.2% (BICAMS-M) of the variance (*p*<.001).

**Conclusion:**

In our clinically stable, small RRMS cohort, BVL/year, thalamic and hippocampal volumes are associated with cognition in contrast to sNfL and sGFAP. EDSS as a global disability score correlates with cognition but adds little beyond MRI. These results require further validation using a prospective design and broader cohort.

**Supplementary Information:**

The online version contains supplementary material available at 10.1186/s12883-026-04894-6.

## Introduction

Cognitive impairment and fatigue are common in multiple sclerosis (MS), affecting 34–65% and up to 95% of patients, respectively, with highest frequency in attention, episodic memory, and information processing speed [1, 2]. Thus, cognitive impairment and fatigue could lead to individual patient’s and economic burden [2]. Neurological disability, including relapse-dependent and independent worsening, is assessed using the Expanded Disability Status Scale (EDSS) [3]. While EDSS primarily reflects physical disability, its representation of cognition and fatigue is only covered marginally [3]. However, cognition does decline with increasing EDSS, raising the question of appropriate biomarkers of these domains.

Recent evidence highlighted the value of specific MRI biomarkers in explaining cognitive decline and fatigue in MS [1]. Cognitive performance was linked to volumetric measures of deep grey matter, especially the thalamus, neocortex, mesial temporal cortex, and hippocampus [4]. Longitudinal MRI, e.g., brain volume loss per year (BVL/year), may also serve as a biomarker for progression independent of relapse activity (PIRA) [5]. However, the role of BVL/year regarding cognition and fatigue in MS patients with no evidence of PIRA (i.e. clinically stable/no relapses) remains unexplored. Fatigue, in contrast, was primarily associated with functional MRI-based structural connectivity in networks linking the thalamus, cortex, basal ganglia, and limbic system [6].

In MS, serum neurofilament light chain (sNfL) and serum glial fibrillary acidic protein (sGFAP) have emerged as markers to differentiate acute inflammation from progressive disease [7]. Several studies reported higher sNfL in cognitively impaired secondary progressive MS patients with and without signs of disease activity, suggesting its relevance for cognitive decline [8–10]. Conversely, multimodal studies in stable relapsing-remitting MS (RRMS) or progressive MS found no association or predictive value of sNfL for cognition or fatigue [8, 11]. Serum GFAP, however, appears unrelated to cognitive decline [8].

Studies incorporating MRI or serum biomarkers mainly employed the Brief International Cognitive Assessment for Multiple Sclerosis (BICAMS-M; 20–30 min) or Symbol Digit Modalities Test (SDMT) to assess cognition in MS [8, 9, 12, 13]. As executive function is also frequently affected [1], we recently introduced TRACK-MS-Revised (TRACK-MS-R), combining SDMT with verbal fluency (Regensburger Wortflüssigkeitstest; RWT) as an ultra-short (4 min) screening tool for cognitive impairment, with a sensitivity of 97.44% versus the gold standard BICAMS-M [14]. There is a lack of studies investigating other cognitive testing/screening tools than the BICAMS-M regarding volumetric MRI analyses and serum biomarkers.

Our aim of this monocentric study was to examine associations of imaging and serum biomarkers and EDSS as a global disability score with cognition and fatigue in a well-defined retrospective RRMS cohort with no evidence of PIRA. Specifically, we addressed: (i) differences in biomarkers between cognitively impaired vs. non-impaired and fatigued vs. non-fatigued patients; (ii) correlations of EDSS with cognition and fatigue compared to thalamic, hippocampal, and whole brain parenchymal volume; and (iii) the associations of cognition and fatigue with volumetric MRI, BVL/year, EDSS, and serum biomarkers.

## Methods

### MS patients

79 MS patients underwent brain MRI, neuropsychological examination (NPE), and EDSS assessment (baseline EDSS) on the same day at the University Hospital of Ulm (Germany) between December 2022 and June 2024. 54 patients had RRMS according to the lately revised McDonald 2024 criteria; 25 patients with primary or secondary progressive MS were excluded [15]. The newly proposed McDonald 2024 criteria were retrospectively applied after carefully reviewing the medical charts (DKE, HT) with radiological, clinical, and cerebrospinal fluid findings, including the patients’ medical (symptom) history. In 33 participants (of the 54 study participants), sNfL and sGFAP levels were measured on the day of NPE/MRI. Exclusion criteria were: relapses and/or glucocorticoid intake within six months before NPE/baseline EDSS, confirmed PIRA, relapse-associated worsening, or other neurological and/or psychiatric disorders potentially explaining cognitive deficits. Demographic data were obtained from medical records. EDSS scores were determined by experienced neurologists (IV, DT, TF) at each visit. The neurologists were not blinded for either medical or clinical records due to the fact that patients were seen in the outpatient or inpatient unit before being retrospectively recruited. For each patient, EDSS scores from three consecutive visits (3, 6, 9, 12, 18, or 24 months before MRI/NPE and baseline EDSS, respectively) were analyzed to exclude PIRA. Again, medical records including the counted EDSS score from each visit in our outpatient or inpatient unit were checked (DKE, HT) for any relapse or disability associated worsening. Disability worsening was defined as an EDSS increase of ≥ 1.0 point for baseline EDSS ≤ 5.5, or ≥ 0.5 points for baseline EDSS > 5.5 [16]. As this was a retrospective study, confirmation intervals varied; however, disability worsening was required to be verified at ≥ 12 and ≥ 24 weeks prior to baseline EDSS (same day as MRI/NPE), as previously suggested [16]. When standardized recordings were unavailable, three time points with a minimum confirmation period of six months were used. Patients remained relapse-free during the confirmation period and at least in the last 90 days before any EDSS assessment.

### Healthy controls

During the same recruitment period as the MS cohort, 76 healthy controls without a history of chronic inflammatory/autoimmune, or neurodegenerative diseases of the central nervous system were enrolled and matched for age, sex, and education. Healthy controls were recruited from the families and colleagues of the authors, local sports centers or by posting notices in the University Hospital of Ulm. To ensure comparability between patients (*n* = 54) and healthy controls (*n* = 76), matching was assessed using t-tests for the continuous variables age and education, and chi^2^-test for sex. No significant group differences were found: Age *t*(128)=–1.078, *p*=.283, years of education *t*(123)=–0.906, *p*=.367, and sex *χ²*(1)=0.06, *p*=.804. Further demographical data of the healthy controls are found in Table S1 (Supplementary Material). Healthy controls received the same neuropsychological assessment as MS patients and were recruited for z-standardization of NPE and self-reported questionnaires (Tables S2+S3, Supplementary Material).

### MRI data acquisition and analysis

Each MRI was performed as part of routine follow-up diagnostics using the same 1.5 T Magnetom Symphony scanner (Siemens Medical, Erlangen, Germany). The protocol included sagittal 3D T2-FLAIR (fluid-attenuated inversion recovery, 176 slices (1.0 mm thickness), echo time = 365 ms, repetition time = 5000.0 ms, in a 448 mm×512 mm matrix) and 3D T1-weighted MPRAGE (magnetization-prepared rapid acquisition with gradient echo) sequences with or without Gadolinium contrast (144 sagittal slices (1.2 mm thickness), echo time = 2.8 ms, repetition time = 1900 ms, in-plane resolution = 1.0 × 1.0 mm, in a 208 mm×256 mm matrix). After acquisition and pseudonymization in accordance with the General Data Protection Regulation (EU 2016/679), MRI data were transferred to jung diagnostics GmbH for automated analysis using Biometrica software (version 1.1). T2-lesion count and volume (ml) were determined from 3D T2-FLAIR and 3D MPRAGE images based on a 3D convolutional neural network (3D-CNN) approach [17]. New or enlarged T2-lesions in follow-up MRIs were also detected automatically using a 3D-CNN approach and verified by an experienced neuroradiologist (KK) [18]. For volumetric analysis, whole brain parenchymal (BP), thalamic (THAL), and hippocampal (HIPP) volumes were segmented automatically from 3D MPRAGE images using a validated CNN-based model [19]. Volumes were normalized to z-scores and adjusted for age and total intracranial volume (TIV) using a reference database of 5059 healthy controls [20]. Longitudinal brain volume loss (BVL) was assessed using “BrainLossNet”, a CNN-based method for automated BVL estimation [21, 22]. BVL (%) was calculated between two 3D MPRAGEs of the same patient taken one year apart (on the same MRI scanner), and the annual BVL rate was derived by dividing by the time interval (years) between those two scans. Rates were age-adjusted based on a healthy cohort of 563 individuals [22]. An age-adjusted BVL <–0.4% per year was considered pathological [22, 23]. MRI activity was defined either for new and/or enlarging T2 lesions or Gadolinium-enhancing T1 lesions.

### NfL and GFAP measurements in serum

For the measurements of NfL and GFAP in serum, we used the microfluidic Ella platform (BioTechne, Minneapolis, MA, USA). Directly upon venipuncture, the samples were centrifuged at 2000 g for 10 min and the extracted serum was aliquoted and frozen within 30 min at − 80 °C. All measurements were performed according to the manufacturer’s instructions. Age-dependent z-scores for NfL and GFAP in serum (Table S4) were calculated as previously described [24, 25].

### Neuropsychological assessment

A trained neuropsychologist (LTB) carried out all neuropsychological assessments under supervision (DL, IU). The assessment included the Verbaler Lern- und Merkfähigkeitstest (VLMT), the Brief Visuospatial Memory Test-Revised (BVMT-R) to evaluate short- and long-term verbal/visual memory in addition to learning ability [26, 27], and the SDMT to assess information processing speed [28]. These tests are part of the well-established gold standard for neuropsychological testing in MS, the BICAMS-M (German version) [29]. Additionally, we administered the previously published TRACK-MS-R screening tool, which comprises the SDMT and the RWT [14]. The RWT is a widely used German tool to measure verbal fluency with the phonematic fluency letter “S” [30]. Fatigue was measured using the Fatigue Scale for Motor and Cognitive Functions (FSMC), distinguishing between motor (FSMC-M) and cognitive (FSMC-C) fatigue categorized as mild (≥ 22 points), moderate (≥ 28 points), or severe (≥ 34 points) [31]. In addition, depression and anxiety were assessed using the Hospital Anxiety and Depression Scale (HADS), a patient-reported measure ranging from 0 to 21 points with 7 items (0–7: none, 8–10: mild, 11–14: moderate, 15–21: severe) [32].

### Ethics

The ethics review committee of the University of Ulm approved this study in accordance with the principles of the Declaration of Helsinki (numbers: 20/10 on 3rd May 2010; 272/23 on 22nd September 2023; 335/23 on 19th December 2023). Written informed consent for inclusion was obtained from each patient including a statement regarding further processing of the data, e.g., publications.

### Statistics

Statistical analyses were performed using IBM^®^ SPSS^®^ Statistics 28.0.1.0 (Armonk, New York, USA) and GraphPad Prism 10.3.0 (La Jolla, CA, USA) for macOS. Continuous variables with non-normal distribution (Shapiro-Wilk test) were reported as median and range; categorical variables as absolute and relative frequencies. Spearman’s rank correlation assessed non-parametric bivariate associations, and group differences were analyzed with the Mann-Whitney U test. Linear regression analyses in separate models were applied to test for associations of different MRI parameters and serum biomarkers with cognitive performance. We assessed multicollinearity in all regression models by examining variance inflation factors (VIF) and tolerance statistics. Statistical significance was assumed for *p*<.05. Z-scores for neuropsychological tests and self-reported questionnaires were calculated as $\:z=\frac{\chi\:\:-\:\mu\:HC}{\:\sigma\:HC}$, with $\:\chi\:$ as the individual patient’s value, and $\:\mu\:HC$ and $\:\sigma\:HC$ as the mean value and standard deviation of the healthy controls (HC). Composite scores of BICAMS-M and TRACK-MS-R were computed by averaging subtest z-scores. Cognitive impairment was defined as z-scores ≤–1.0. This stringent cut-off was applied due to following widely used conventions in clinical neuropsychology [33]. According to this, scores between − 1 and − 2 SD are considered moderately cognitively impaired and scores within ± 1 SD of the mean are considered average [33].

## Results

### Patient characteristics

Table 1 summarizes the main demographic and clinical characteristics of the cohort. Most patients were female (*n* = 38; 70.4%), with a median age of 39.6 years (range 18.2–73.1) and a median baseline EDSS of 2.0 (range 0.0–6.5). No patients exhibited PIRA, though 11 (20.4%) showed MRI activity. The median interval between MRIs was 1.0 years (range 0.28–3.82). Eleven patients experienced a relapse within the 12 months preceding neuropsychological assessment, but > 90 days before each counted EDSS and during the confirmation period of at least 6 months. 20 (37.1%) MS patients were on high-efficacy therapy. In general, 26 (48.1%) patients had their first disease-modifying drug and 23 (42.6%) were on escalating/high-efficacy therapy. Only 1 (1.9%) patient was switched to de-escalating therapy. Four (7.4%) patients had no disease-modifying therapy. Cognitive impairment was observed in 21 (38.9%) patients per BICAMS-M and 19 (35.2%) per TRACK-MS-R. Moderate/severe motoric and cognitive fatigue were reported in 30 (55.6%) and 31 (57.4%) patients, respectively. 42 (77.8%) MS patients had no depression and 39 (72.2%) no anxiety according to HADS, whereas 8 (14.8%) patients had mild depression/anxiety, 4 (7.4%) and 6 (11.1%) patients had moderate depression and anxiety, respectively, and only 1 (1.9%) patient had severe anxiety. According to HADS, no MS patient had severe depression. Only 5 (9.3%) MS patients took antidepressant medication. Raw data and z-scores of the NPE for patients and healthy controls are provided in Tables S2–S4 (Supplementary Material).

**Table 1:** Main demographical and clinical characteristics at time of MRI, NPE, and baseline EDSS assessment

Variable	RRMS (*n* = 54)
Sex, n (f/m) (%)	38/16 (70.4/29.6)
Age, y, *M* (range)	39.62 (18.15–73.10)
Years of education, y, *M* (range)	14.5 (8.0–23.0)
Time since onset to NPE/MRI, mo, *M* (range)	96.0 (11.0-300.0)
Disease-modifying therapy use, n (%)	50 (92.6)
-None, n (%)	4 (7.4)
-Dimethylfumarat, n (%)	8 (14.8)
-Diroximelfumarat, n (%)	3 (5.6)
-Teriflunomid, n (%)	4 (7.4)
-Interferon beta-1a, n (%)	3 (5.6)
-Glatirameracetat, n (%)	3 (5.6)
-Fingolimod, n (%)	5 (9.3)
-Ozanimod, n (%)	2 (3.7)
-Cladribin, n (%)	2 (3.7)
-Natalizumab, n (%)	6 (11.1)
-Ocrelizumab, n (%)	9 (16.7)
-Ofatumumab, n (%)	5 (9.3)
Relapse in last 12 months^a^, n, (%)	11 (20.5)
Motoric fatigue, +(n)/-(n), (%)	30/24 (55.6/44.4)
Cognitive fatigue, +(n)/-(n), (%)	31/23 (57.4/42.6)
CI in BICAMS-M, +(n)/-(n), (%)	21/33 (38.9/61.1)
CI in TRACK-MS-R, +(n)/-(n), (%)	19/35 (35.2/64.8)
HADS-D, *M* (range)	3.5 (0.0–14.0)
-none, n (%)	42 (77.8)
-mild, n (%)	8 (14.8)
-moderate, n (%)	4 (7.4)
-severe, n (%)	-
HADS-A, *M* (range)	6.0 (0.0–15.0)
- none, n (%)	39 (72.2)
- mild, n (%)	8 (14.8)
- moderate, n (%)	6 (11.1)
- severe, n (%)	1 (1.9)
EDSS at baseline, *M* (range)	2.0 (0.0-6.5)

*f* female, *m* male, *y* year, *M* median, *NPE* Neuropsychological examination, *mo* month, *EDSS* Expanded disability status scale, *CI* Cognitive impairment, *BICAMS-M* German version of the “Brief International Cognitive Assessment for Multiple Sclerosis”, *HADS-D* Hospital Anxiety and Depression Scale-Depression, *HADS-A* Hospital Anxiety and Depression Scale-Anxiety

^a^Relapse-free during the confirmation period (baseline EDSS and two further EDSS measurements at ≥ 12 and ≥ 24 weeks since baseline EDSS), no relapse associated worsening in the last 90 days before any EDSS count

### Group comparisons regarding cognition and fatigue

**Fig. 1:**
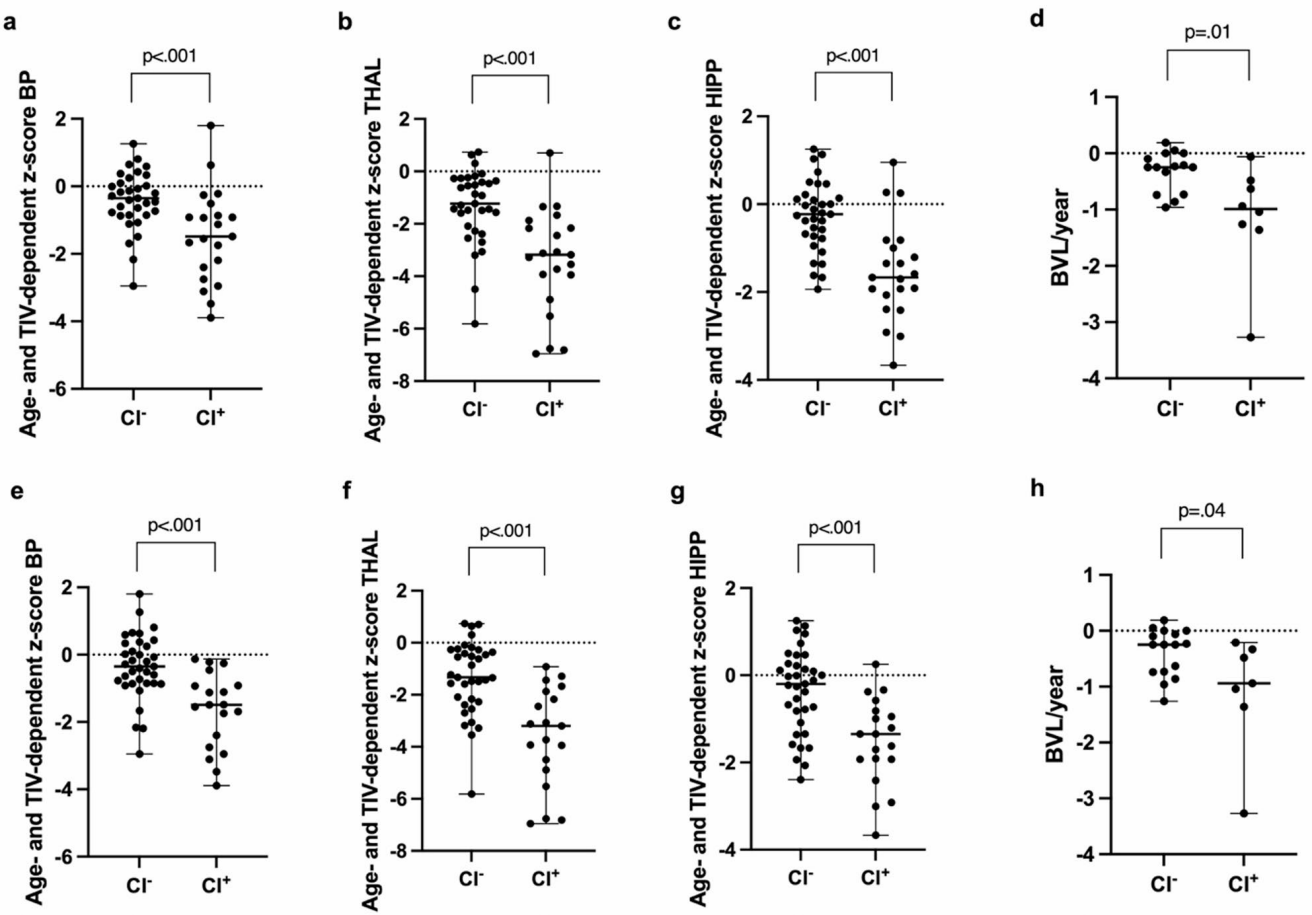
Comparison of cognitively impaired (CI^+^) and non-impaired (CI^–^) PIRA^–^ RRMS patients regarding MRI volumetrics. We performed group comparisons using Mann-Whitney U test between PIRA^–^ RRMS patients with (CI^+^, *n* = 21) and without cognitive impairment (CI^–^, *n* = 33) according to the BICAMS-M (**a-d**) and TRACK-MS-R (**e-h**; CI^+^: *n* = 19; CI^–^: *n* = 35). **a**;** e** age- and TIV-dependent z-score for BP, (**b**;** f**) age- and TIV-dependent z-score for THAL, (**c**;** g**) age- and TIV-dependent z-score for HIPP, and (**d**;** h**) BVL/year. Values are displayed with median and range. P-values < 0.05 were considered as statistically significant. PIRA: progression independent of relapse activity, PIRA^–^: no evidence of PIRA, RRMS: relapsing remitting multiple sclerosis, MRI: magnetic resonance imaging, TIV: total intracranial volume, BP: whole brain parenchymal volume, THAL: thalamus volume, HIPP: hippocampal volume, BVL: brain volume loss, BICAMS-M: German version of the “Brief International Cognitive Assessment for Multiple Sclerosis”

**Fig. 2:**
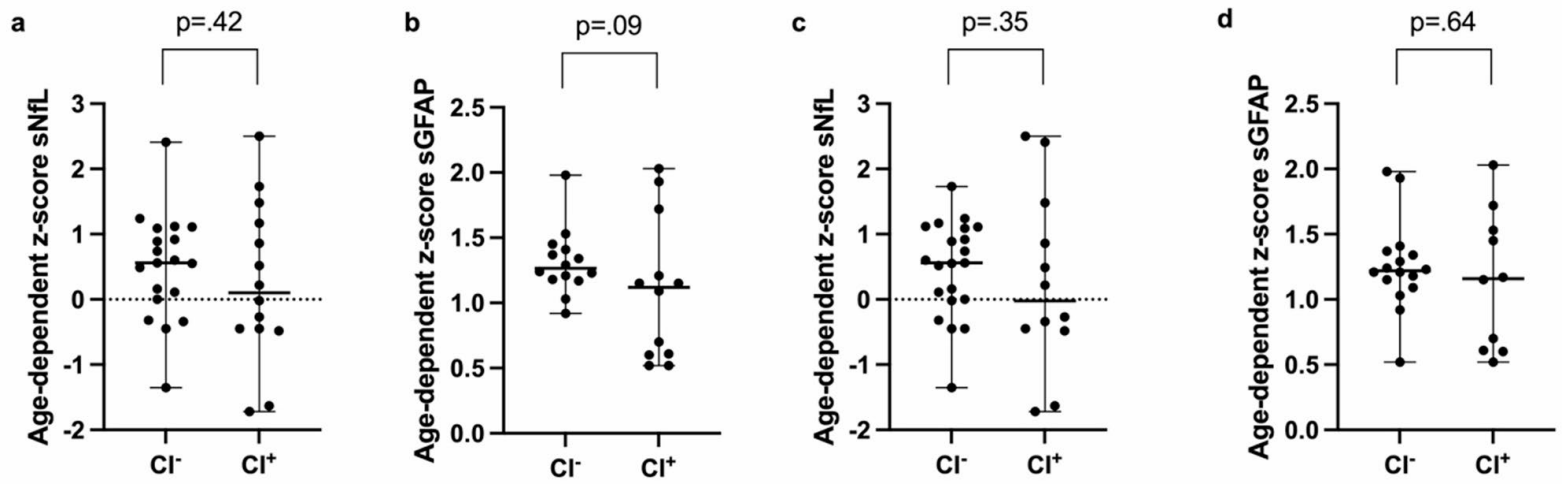
Comparison of cognitively impaired (CI^+^) and non-impaired (CI^–^) PIRA^–^ RRMS patients: sNfL and sGFAP. We performed group comparisons using Mann-Whitney U test between PIRA^–^ RRMS patients with (CI^+^, *n* = 21) and without cognitive impairment (CI^–^, *n* = 33) according to the BICAMS-M (**a** and **b**) and TRACK-MS-R (**c** and **d**; CI^+^: *n* = 19; CI^–^: *n* = 35). **a** and **c** age-dependent z-score for sNfL, (**b** and **d**) age-dependent z-score for sGFAP. Values are displayed with median and range. P-values < 0.05 were considered as statistically significant. PIRA: progression independent of relapse activity, PIRA^–^: no evidence of PIRA, RRMS: relapsing remitting multiple sclerosis, BICAMS-M: German version of the “Brief International Cognitive Assessment for Multiple Sclerosis”, sNfL: serum neurofilament light chain, sGFAP: serum glial fibrillary acidic protein

MS patients with cognitive impairment according to BICAMS-M showed significantly lower thalamic, hippocampal, and whole-brain parenchymal volumes (all *p*<.001) and higher BVL/year (*p*=.01) compared to non-impaired patients (Fig. 1). No significant group differences were observed for sGFAP (BICAMS-M: *p*=.09) or sNfL (BICAMS-M: *p*=.42, Fig. 2). Similarly, volumetric MRI measures (*p*>.09; Figure S1 in Supplementary Material) and serum biomarkers (*p*>.07; Figure S2 in Supplementary Material) did not differ between patients with no/mild versus moderate/severe motoric or cognitive fatigue. Our recently proposed ultra-short cognitive screening tool TRACK-MS-R showed similar findings compared to BICAMS-M regarding volumetric MRI measures (BP/THAL/HIPP: *p*<.001; BVL/year: *p*=.04), sGFAP (*p*=.64), and sNfL (*p*=.35). Graphs are displayed in Figs. 1e-h and 2c + d.

### Correlation of cognition, fatigue, disability, MRI, and serum biomarkers

**Fig. 3:**
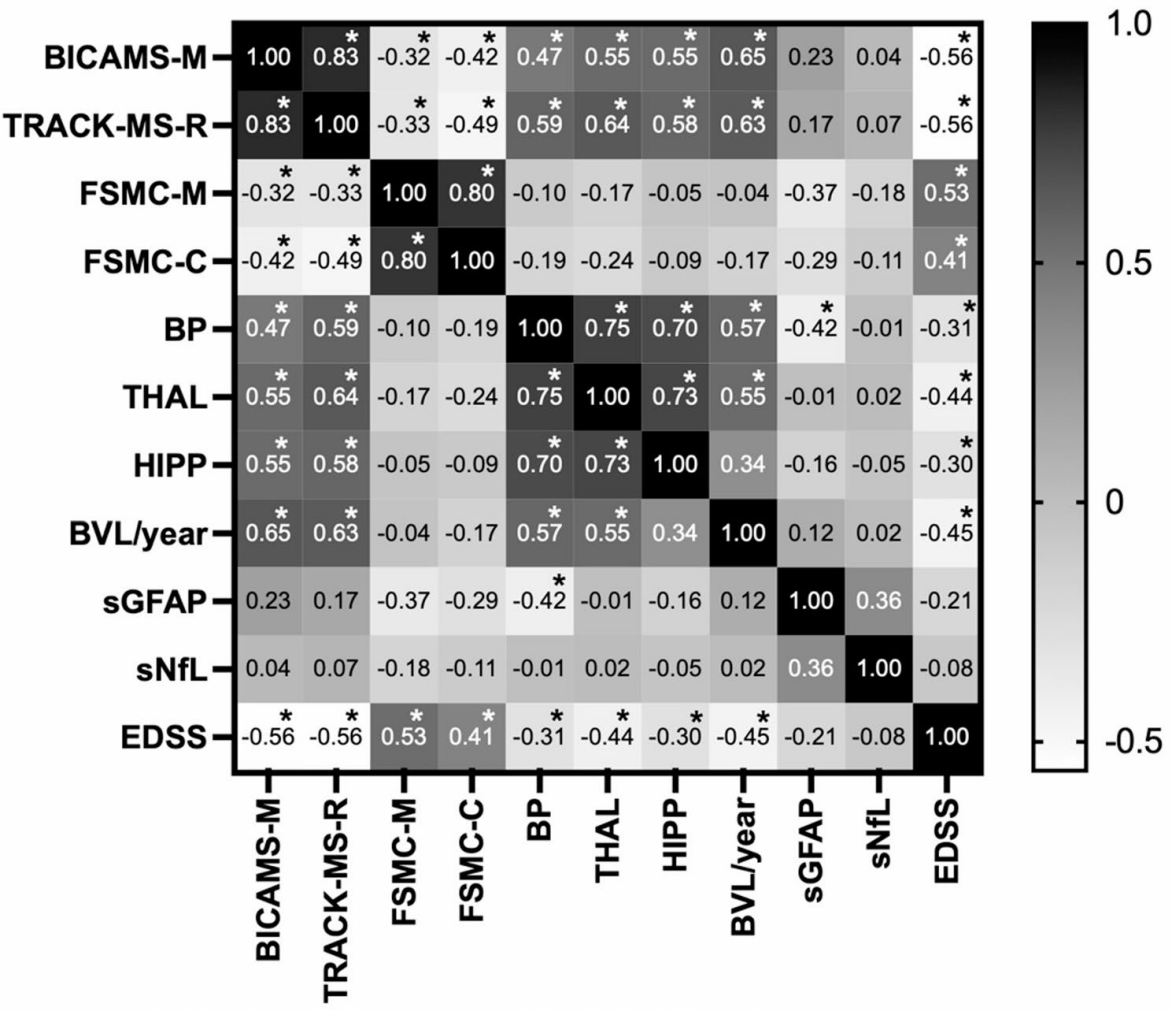
Correlation matrix of the investigated MRI, laboratory, disability and neuropsychological variables. We performed correlation analyses for cognition (BICAMS-M and TRACK-MS-R), fatigue (FSMC-M and FSMC-C), volumetric MRI measures (BP, THAL, HIPP, BVL/year), serum biomarkers (sGFAP and sNfL), and disability (EDSS) in 54 RRMS patients. Displayed values represent Spearman’s rank correlation coefficients (*r*), with 1.0 shown in black and − 0.6 in white. Color intensity indicates the strength of correlation, regardless of direction. Only complete variable pairs were included. No correction for multiple comparisons was applied. Asterisks (*) indicate statistically significant correlations (*p*<.05). BICAMS-M: Brief International Cognitive Assessment for Multiple Sclerosis, FSMC-M: Fatigue Scale for Motor and Cognitive Functions (motor fatigue), FSMC-C: Fatigue Scale for Motor and Cognitive Functions (cognitive fatigue), EDSS: expanded disability status scale, BP: whole brain parenchymal volume, THAL: thalamus volume, HIPP: hippocampal volume, BVL: brain volume loss, sGFAP: serum glial fibrillary acidic protein, sNfL: serum neurofilament light chain

In correlation analyses (Fig. 3) using Spearman’s rank coefficient *r*, BICAMS-M showed moderate to strong significant correlations (all *p*<.001) with thalamic (*r*=.55), hippocampal (*r*=.55), and whole brain parenchymal volumes (*r*=.47), as well as BVL/year (*r*=.65). EDSS correlated negatively with BICAMS-M (*r*=–.56; *p*<.001). Fatigue correlated moderately with BICAMS-M (FSMC-M: *r*=–.32; *p*=.02; FSMC-C: *r*=–.42; *p*=.002). EDSS also correlated with fatigue (FSMC-M: *r*=.53; *p*<.001; FSMC-C: *r*=.41; *p*=.002) and showed moderate negative correlations with volumetric MRI measures (BP: *r*=–.31; *p*=.02; THAL: *r*=–.44; *p*<.001; HIPP: *r*=–.30; *p*=.03; BVL/year: *r*=–.45; *p*=.03). No significant correlations were found between BICAMS-M, fatigue, or EDSS with sNfL and sGFAP (*p*>.13), although sGFAP correlated moderately with whole brain parenchymal volume (*r*=–.42; *p*=.03). The findings regarding BICAMS-M are supported by the correlation analyses of the different MRI metrics, serum biomarkers, fatigue, and our newly proposed ultra-short cognitive screening tool TRACK-MS-R. Additionally, the respective *r*- and *p*-values can be found in Fig. 3.

### Association of MRI metrics, sNfL, and sGFAP with cognitive performance

Multiple linear logistic regression analyses in separate models due to strong collinearity of BP (VIF = 10.7, tolerance 0.09) and THAL (VIF = 7.2, tolerance 1.4) identified several significant associations with cognitive performance, as assessed by the BICAMS-M composite score (Table 2). Global disability (EDSS) was negatively associated with cognitive performance (BICAMS-M: β=–0.444; *t*(52)=–4.080; *p*<.001), explaining 24.6% of the respective score variance. EDSS was also associated with fatigue, accounting for 21.5% of the variance in motoric fatigue (β = 0.475; *t*(52) = 3.738; *p*<.001) and 13.7% in cognitive fatigue (β = 0.381; *t*(52) = 2.842; *p*=.006). Brain volumetric z-scores were significantly associated with cognitive performance. BVL/year explained the largest variance in BICAMS-M (63.2%). Thalamic volume accounted for 28.5% of the variance in BICAMS-M, while hippocampal volume explained 31.8% and 27.5%, respectively. Whole-brain parenchymal volume was also associated with cognitive impairment (BICAMS-M: 23.1%). Serum biomarkers sGFAP and sNfL showed no significant associations with cognitive scores (all *p*>.08). Linear regression analyses were also conducted with TRACK-MS-R and the different MRI metrics, serum biomarkers, and EDSS. We found similar significant associations for BP (30.3%; *p*<.001), HIPP (27.5%; *p*<.001), THAL (31.2%; *p*<.001), BVL/year (61.8%; *p*<.001), and EDSS (27.7%; *p*<.001) as displayed in Table 2.

**Table 2:** Associations of volumetric MRI data, sNfL, sGFAP, and EDSS with cognition and fatigue in RRMS (*n* = 54)

Variable	BICAMS-M composite score	TRACK-MS- *R* composite score	z-score FSMC-M^c^	z-score FSMC-C^c^
EDSS	(β=–0.444, *t*(52)=–4.080, *p*<.001)	(β=–0.356, *t*(52)=–4.415, *p*<.001)	(β = 0.475, *t*(52) = 3.738, *p*<.001)	(β = 0.381, *t*(52) = 2.842, *p=*.006)
z-score BP^a^	(β = 0.572, *t*(52) = 3.955, *p*<.001)	(β = 0.495, *t*(52) = 4.749, *p*<.001)	*p=*.377	*p=*.133
z-score THAL^a^	(β = 0.396, *t*(52) = 4.552, *p*<.001)	(β = 0.313, *t*(52) = 4.851, *p*<.001)	*p=*.473	*p=*.252
z-score HIPP^a^	(β = 0.722, *t*(52) = 4.923, *p*<.001)	(β = 0.508, *t*(52) = 4.439, *p*<.001)	*p=*.88	*p=*.717
BVL/year	(β = 1.452, *t*(21) = 6.007, *p*<.001)	(β = 1.053, *t*(21) = 5.834, *p*<.001)	*p=*.479	*p=*.299
z-score sGFAP^b^	*p=*.651	*p=*.431	*p=*.08	*p=*.17
z-score sNfL^b^	*p=*.862	*p=*.878	*p=*.347	*p=*.679

*BICAMS-M* German version of the “Brief International Cognitive Assessment for Multiple Sclerosis”, FSMC-M: Fatigue Scale for Motor and Cognitive Functions (motor fatigue), *FSMC-C* Fatigue Scale for Motor and Cognitive Functions (cognitive fatigue), *EDSS* Expanded disability status scale, *BP* whole brain parenchymal volume, *THAL* Thalamus volume, *HIPP* Hippocampal volume, *BVL* Brain volume loss, *sGFAP* Serum glial fibrillary acidic protein, *sNfL* serum neurofilament light chain

^a^Age- and total-intracranial-volume-dependent

^b^Age-dependent

^c^Z-standardization using age-, sex-, and education-matched healthy controls (n = 76) as described in the “Methods” section

### Group comparisons of clinically stable MS patients with and without MRI activity

Group comparisons between RRMS patients with (*n* = 11) and without MRI activity (*n* = 41) using the Mann-Whitney U test found no significant differences regarding cognition, MRI volumetrics, or serum biomarkers (*p* >.36). All data are found in Figure S3 (Supplementary Material).

## Discussion

Our study provided new insights into the associations between volumetric MRI metrics, serum biomarkers, cognition, fatigue, and disability (EDSS) incorporating the concept of PIRA in RRMS.

Among clinically stable RRMS patients with mild physical disability (median EDSS = 2.0), up to 38.9% showed cognitive impairment according to BICAMS-M and the newly proposed ultra-short TRACK-MS-R screening tool, which incorporates executive functions in addition to information processing speed. This prevalence aligns with previous reports, although cognitive impairment was defined more stringent in our study as z-score ≤–1.0 [11]. No associations were found between serum NfL/serum GFAP or volumetric MRI parameters and fatigue, consistent with a recent systematic review reporting significant links between fatigue and thalamic atrophy in only 5 of 48 studies [6]. Instead, fatigue was more closely related to altered resting-state functional connectivity, activation, and dynamics within cortico-thalamo-basal-ganglial, fronto-striatal, and limbic networks, potentially reflecting myelin and/or axonal damage [6]. Fatigue in multiple sclerosis is increasingly conceptualized as a network-level phenomenon rather than a consequence of focal structural atrophy. In a resting-state fMRI study by Jaeger et al., especially the superior ventral striatum functional connectivity was reduced suggesting impairment in motor functions, attention, and reward regulation [34]. Together with the positive correlation between the functional connectivity of the dorsolateral prefrontal cortex and fatigue severity with the parietal operculum and supramarginal gyrus in fatigued MS patients, this could reflect an ongoing and maladaptive process of reactions on incoming stimuli contributing to an effort-reward imbalance [34]. Overactivation of the dorsolateral prefrontal cortex was associated with fatigue in former studies [35, 36].

In line with prior studies, sNfL, a marker of axonal injury/loss, showed no correlation or association with respect to self-reported fatigue, neuropsychological performance, or volumetric MRI measures in our clinically stable RRMS cohort [11, 37]. But due to the small sample size of this subgroup analysis attributable to the retrospective design of our study, these findings should be interpreted with caution. Previous longitudinal studies showed that baseline sNfL, but not sGFAP, predicts future cognitive decline in SDMT and Wechsler Abbreviated Scale of Intelligence after median follow-ups of 3.1 and 2 years, respectively [8, 10]. In the present cohort, the only serum–MRI association was observed between sGFAP and whole brain parenchymal volume, in agreement with previous findings. Elevated sGFAP levels were typically associated with progressive, non-active MS characterized by disability accumulation and grey matter atrophy, whereas sNfL was more closely related to acute inflammatory MRI activity and clinical relapses [38].

In our study, cognitive impairment correlated with thalamic, hippocampal, and whole brain parenchymal volumes. Thalamic atrophy has become a major focus of neuroimaging research in MS, as its volume loss occurs independently of disease subtype and can already be detected at disease onset in young patients presenting with initial clinical symptoms or radiologically isolated syndrome [39]. Its role as an information relay center renders it a pivotal component in terms of cognition in MS [39]. Thalamic and hippocampal atrophy were directly linked to deficits in information processing speed and episodic memory, as well as their severity [40, 41]. Through its anatomical composition of different nuclei groups and functional division, the thalamus is linked to different loci for cognitive domains, e.g. prefrontal cortex, limbic system, and the parietal lobe [42, 43]. Moreover, 52.7% of the variance in information processing speed and executive function performance were explained by thalamic volume together with increased posterior thalamic activation on fMRI [44], partially consistent with our findings for TRACK-MS-R and thalamic volume. For this combined score of information processing speed (SDMT) and executive function (RWT), we observed predictive value explaining up to 31.2% of the variance and a strong-to-moderate correlation with all volumetric MRI parameters. Annualized brain volume loss (BVL/year), accounting for up to 63.2% of the variance in BICAMS-M and TRACK-MS-R, was strongly associated with cognitive performance. BVL/year was recently proposed as a potential marker of short-term disability progression in a large multicenter cohort of 453 relapse- and new/enlarged T2-lesion-free RRMS patients [5]. Notably, no relevant multicollinearity was observed for annualized brain volume loss (VIF = 1.6, tolerance 0.65) in our small RRMS cohort, indicating that the observed association was not driven by collinearity with other model variables. Our findings support the important role of BVL/year for neurodegenerative change, emphasizing its role in cognitive impairment independent of inflammatory activity in clinically stable RRMS.

Another key finding of our study was that global disability (EDSS), despite remaining stable for up to 24 months in some cases under the PIRA concept, correlated with cognitive impairment and all volumetric MRI measures. Besides cognition, the EDSS also correlated moderately to fatigue in contrast to MRI measures and serum NfL and GFAP. There is a matter of debate whether the EDSS captures cognitive impairment and fatigue adequately as it provides only a broad estimate of these deficits/symptoms and should be rather interpreted as a global disability score [3]. Only two studies evaluated cognitive progression independent of disability worsening so far [12, 13]. Ziccardi et al. reported that 80.4% of RRMS patients with cognitive decline on the SDMT showed progression independent of clinical relapse or MRI activity (PIRMA) regarding cognitive worsening [13], consistent with Fuchs et al., who focused only on physical disability/relapses (PIRA) [12]. The latter introduced the term “cognitive PIRA” for 89% of their RRMS cohort [12]. In contrast, transient cognitive worsening may be linked to focal inflammatory MRI lesions as an “isolated cognitive relapse” [45]. This points to the limitations of our study.

We included the thalamus and hippocampus in our correlation and regression analyses on cognition and fatigue due to the abundance of literature linking their atrophy in MS patients with cognitive deficits. However, since TRACK-MS-R was suggested as a promising ultra-short screening tool incorporating verbal fluency as an important component of executive functions, other regions may also contribute to cognitive impairment. Thus, we cannot exclude that microstructural MRI patterns in other regions better reflect cognition and fatigue in MS. Another limitation was the absence of MRI data in healthy controls, as this retrospective cohort was primarily recruited for z-standardization of TRACK-MS-R [14].

The MS cohort was small (*n* = 54) and heterogeneous regarding disease-modifying treatment, with missing sNfL and sGFAP data in 21 patients (38.8%) due to the retrospective design and sparce biosample availability. This design also caused disparities in group sizes, e.g., group comparisons in clinically stable patients with/without MRI activity since the last MRI one year prior to NPE. Missing data were handled automatically in SPSS using pairwise exclusion to maximize valid observations per variable. But the small sample size and the limited availability of serum NfL and GFAP data reduced statistical power, particularly in subgroup analyses, increasing the likelihood of type II error. Therefore, conclusions regarding the lack of associations for sNfL and sGFAP should be interpreted cautiously.

Another limitation is that the neurologists in the inpatient and outpatient units who examined the patients for PIRA were not blinded. However, recruitment was retrospective. Additionally, no correction was made for depression which could have affected cognition. The prevalence for depression in our RRMS cohort was 22.2% (12 patients) and slightly higher than reported in a recent meta-analysis (15.78%) [46]. But in contrast to this meta-analysis, no patient in our cohort suffered from severe depression with 4 patients having moderate and 12 patients having mild depression, respectively. Depression does affect cognition, but the effect size is higher the more pronounced the depressive symptomatology is [47]. However, we cannot exclude an impact of depression on cognition and/or fatigue.

As expected due to the high correlations (*r*>.7), MRI volumetric measures showed substantial intercorrelations with elevated VIF values, particularly for thalamic (VIF = 10.7) and brain parenchymal volume (VIF = 7.2). Given strong intercorrelations, each MRI parameter and blood-based biomarker was entered into separate models and converted to z-scores. While the overall model remained stable, this degree of multicollinearity may have affected the precision and interpretability of individual regression coefficients for correlated MRI metrics. Therefore, results for single MRI volumetric measures should be interpreted with caution and should be investigated in a larger (prospective) cohort. Due to the small sample size, the relatively high proportion of explained variance for some parameters, e.g., BVL/year, should not be interpreted as evidence of robust predictive utility, but rather as an association observed in this specific sample that warrants validation in larger, independent cohorts. Although no relevant multicollinearity was detected, the magnitude of the explained variance may nevertheless reflect, at least in part, model instability or inflation of regression coefficient. Our analyses were conducted within an exploratory framework and multiple correlations and regression analyses were performed without formal correction for multiple testing. Therefore, our findings should be interpreted with caution, conversely, the applied parametric methods, e.g., linear regression analyses, are generally considered robust.

A major strength was exclusion of confirmed PIRA in the study population. However, we could not apply the composite PIRA definition (including 9-hole-peg-test and timed 25-foot-walk) due to limited data.

## Conclusion

In conclusion, the moderate-to-strong correlations and associations of thalamus, hippocampus, and whole brain parenchymal volume suggest that these MRI biomarkers play a pivotal role in cognitive deficits in MS, even without evidence of PIRA. The association of EDSS with cognition supports its role as a score for “global disability”. Serum biomarkers lacked sufficient power to distinguish cognitively impaired from non-impaired clinically stable RRMS patients. Neither volumetric MRI nor sNfL/sGFAP appeared relevant for MS fatigue in our small RRMS cohort without evidence of PIRA. These data support systematic cognitive screening alongside longitudinal volumetry in clinically stable RRMS and motivate prospective, adequately powered studies that test cognitive outcomes against BVL/year and evaluate the concept of cognitive PIRA. As could be seen in our small clinically stable MS cohort, up to 38.9% of the patients showed cognitive impairment and > 50% were either cognitively or physically fatigued. Phase III trials for potential disease-modifying drugs in MS primarily focus on clinical relapses as primary endpoints. As recently discussed, the question arises as to whether cognition and fatigue could be considered as secondary endpoints, possibly as cognitive PIRA [48].

## Supplementary Information

Supplementary Material 1.

## Data Availability

Anonymized raw data will be made available on reasonable request. But generally, all data analyzed during this study are included in this published article and its supplementary information files.

## Abbreviations

3D-CNN: 3D convolutional neural network
BICAMS-M: Brief International Cognitive Assessment for Multiple Sclerosis
BP: Brain parenchymal volume
BVL/year: Brain volume loss per year
BVMT-R: Brief Visuospatial Memory Test-Revised
CI: Cognitive impairment
EDSS: Expanded Disability Status Scale
f: female
FLAIR: Fluid-attenuated inversion recovery
FSMC: Fatigue Scale for Motor and Cognitive Functions
FSMC-C: Fatigue Scale for Motor and Cognitive Functions (cognitive)
FSMC-M: Fatigue Scale for Motor and Cognitive Functions (motoric)
HIPP: Hippocampal volumes
m: male
M: Median
mo: month
MPRAGE: Magnetization-prepared rapid acquisition with gradient echo
MRI: Magnetic resonance imaging
MS: Multiple sclerosis
n: Count
NPE: Neuropsychological examination
PIRA: Progression independent of relapse activity
r: Spearman’s rank
RRMS: Relapsing-remitting multiple sclerosis
RWT: Regensburger Wortflüssigkeitstest
SD: Standard deviation
SDMT: Symbol Digit Modalities Test
sGFAP: Serum glial fibrillary acidic protein
sNfL: Serum neurofilament light chain
THAL: Thalamic volume
TIV: Total intracranial volume
TRACK-MS-R: TRACK-MS-Revised
VLMT: Verbaler Lern- und Merkfähigkeitstest
y: year
