# Supplementary material for: Cognition and fatigue in clinically stable multiple sclerosis: EDSS and MRI metrics outperform serum biomarkers
Source: BMC Neurol. 2026 Apr 17;26:260. doi: 10.1186/s12883-026-04894-6 (PMC13094158; doi:10.1186/s12883-026-04894-6)
Supplement: Supplementary file 1 — Supplementary Material 1. [file 12883_2026_4894_MOESM1_ESM.pdf]

# Cognition and Fatigue in Clinically Stable Multiple Sclerosis: EDSS and MRI Metrics Outperform Serum Biomarkers

Deborah K. Erhart[1]*, Luisa T. Balz[1], Roland Opfer[2], Lothar Spies[2], Franziska Bachhuber[1], Ioannis Vardakas[1], Daniela Taranu[1], Stefanie Jung[1], Tanja Fangerau[1], Makbule Senel[1], Kornelia Kreiser[3], Ingo Uttner[1], Dorothée Lulé[1], Hayrettin Tumani[1]*

[1]Department of Neurology, University Hospital Ulm, Oberer Eselsberg 45, 89081 Ulm, Germany
[2]jung diagnostics GmbH, Röntgenstrasse 24, 22335 Hamburg, Germany
[3]Department of Neuroradiology, University Hospital Ulm, Oberer Eselsberg 45, 89081 Ulm, Germany
*Corresponding authors: deborah.erhart@uni-ulm.de; hayrettin.tumani@uni-ulm.de

## Supplementary Material

**Table S1 Demographic data of healthy controls (n=76).**

| **Variable** | Healthy controls (n=76) |
|---|---|
| Gender, n (f/m) (%) | 55/21 (72.4/27.6) |
| Age, y, *M* (range) | 43.0 (19.0-64.0) |
| Years of education, y, *M* (range) | 14.5 (10.0-23.0) |

f= female, m= male, y= year, *M*= median.

**Table S2 Data of neuropsychological assessment and fatigue of RRMS patients (n=54) in raw values.**

| **Neuropsychological subtest/PROM** | RRMS (n=54) |
|---|---|
| Verbal Learning Memory Test (VLMT) - total, *M* (range) | 53.5 (19.0-71.0) |
| Brief Visuospatial Memory Test-Revised (BVMT-R) - total, *M* (range) | 29.0 (1.0-36.0) |
| Symbol Digit Modalities Test (SDMT), *M* (range) | 54.0 (18.0-83.0) |
| Regensburger Wortflüssigkeitstest (RWT), *M* (range) | 18.5 (6.0-39.0) |
| Fatigue Scale for Motor and Cognitive Functions (FSMC) - Motor Fatigue, *M* (range) | 30.0 (10.0-50.0) |
| Fatigue Scale for Motor and Cognitive Functions (FSMC) - Cognitive Fatigue, *M* (range) | 31.0 (10.0-50.0) |

*M*= median, PROM: patient reported outcome measurement.

**Table S3 Data of neuropsychological assessment and fatigue in raw values (Healthy controls, n=76).**

| **Neuropsychological subtest/PROMs** | Healthy controls (n=94) |
|---|---|
| Verbal Learning Memory Test (VLMT) - total, *M* (range) | 61.0 (34.0-73.0) |
| Brief Visuospatial Memory Test-Revised (BVMT-R) - total, *M* (range) | 30.0 (13.0-36.0) |
| Symbol Digit Modalities Test (SDMT), *M* (range) | 61.0 (43.0-83.0) |
| Regensburger Wortflüssigkeitstest (RWT), *M* (range) | 25.0 (11.0-47.0) |
| Fatigue Scale for Motor and Cognitive Functions (FSMC) - Motor Fatigue, *M* (range) | 16.5 (.0-44.0) |
| Fatigue Scale for Motor and Cognitive Functions (FSMC) - Cognitive Fatigue, *M* (range) | 17.0 (.0-42.0) |

*M*= median, PROM: patient reported outcome measurement.

**Table S4 MRI and neuropsychological data incl. serum biomarkers of RRMS patients after z-standardization.**

| **Variable** | RRMS (n=54) |
|---|---|
| Verbal Learning Memory Test (VLMT) - total, *M* (range) | –1.01 (–5.81-1.43) |
| Brief Visuospatial Memory Test-Revised (BVMT-R) - total, *M* (range) | .02 (–5.63-1.44) |
| Symbol Digit Modalities Test (SDMT), *M* (range) | –.77 (–4.70-2.39) |
| Regensburger Wortflüssigkeitstest (RWT), *M* (range) | –.92 (–2.72-2.04) |
| Fatigue Scale for Motor and Cognitive Functions (FSMC) - Motor Fatigue, *M* (range) | 1.62 (–1.11-4.35) |
| Fatigue Scale for Motor and Cognitive Functions (FSMC) - Cognitive Fatigue, *M* (range) | 1.77 (–1.15-4.41) |
| BICAMS-M composite score, *M* (range) | –.52 (–4.65-1.20) |
| TRACK-MS-R, composite score, *M* (range) | –.82 (–3.71-1.29) |
| BP, *M* (range) | –.68 (–3.89-1.8) |
| THAL, *M* (range) | –1.63 (–6.95-.74) |
| HIPP, *M* (range) | –.71 (–3.67-1.25) |
| BVL/year, *M* (range) | –.34 (–3.27-.19) |

| sGFAP, *M* (range) | 1.21 (.52-2.03) |
|---|---|
| sNfL, *M* (range) | .52 (–1.72-2.5) |

BICAMS-M: German version of the “Brief International Cognitive Assessment for Multiple Sclerosis”, BP: whole brain parenchymal volume, THAL: thalamus volume, HIPP: hippocampal volume, BVL: brain volume loss, sGFAP: serum glial fibrillary acidic protein, sNfL: serum neurofilament light chain, *M*= median.

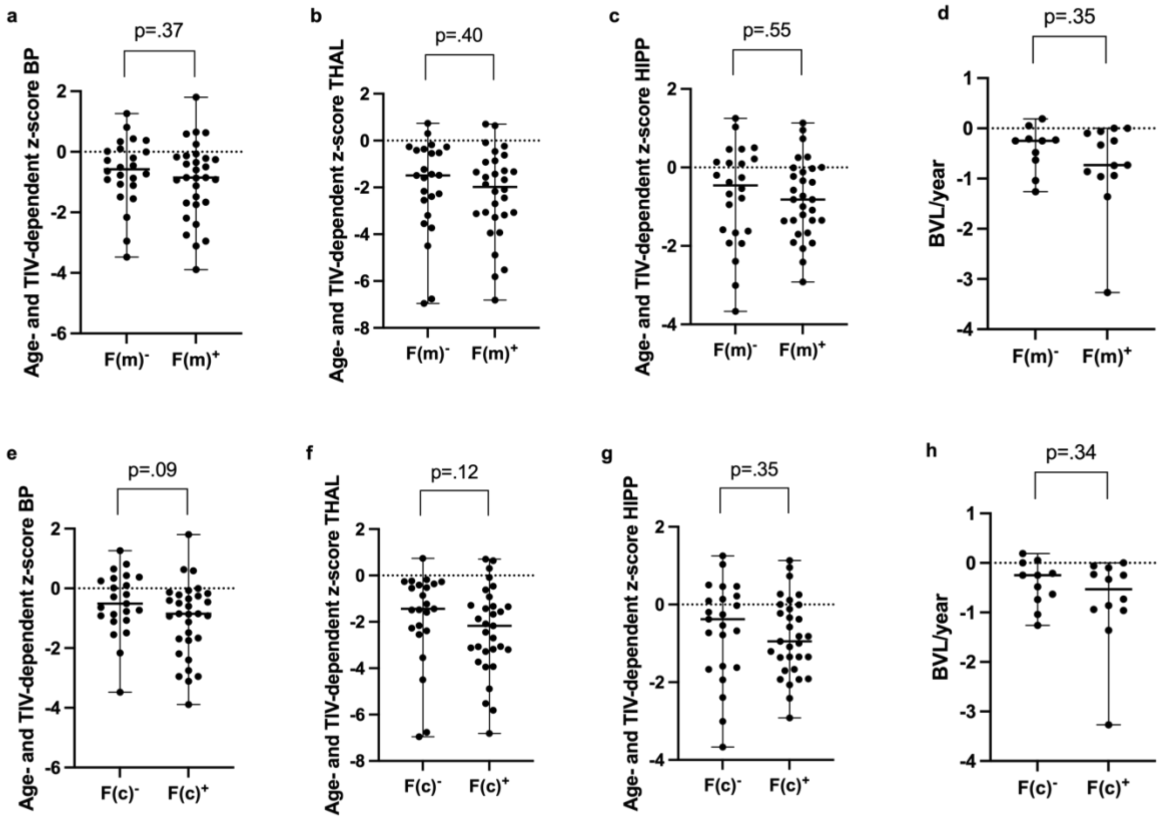


**Figure S1 Group comparisons of clinically stable RRMS (PIRA⁻) patients with (F(m)⁺/F(c)⁺) and without (F(m)⁻/F(c)⁻) motoric (F(m)) or cognitive (F(c)) fatigue regarding volumetric MRI measurements.**

We performed group comparisons using Mann-Whitney-U test between clinically stable RRMS (PIRA⁻) patients with (F(m)⁺, n=30; F(c)⁺, n=31) and without motoric or cognitive fatigue (F(m)⁻, n=24; F(c)⁻, n=23) according to the FSMC-M (**a-d**) and FSMC-C (**e-h**). (**a; e**) age- and TIV-dependent z-score for BP, (**b; f**) age- and TIV-dependent z-score for THAL, (**c; g**) age- and TIV-dependent z-score for HIPP, and (**d; h**) BVL/year. Values are displayed with median and range. P-values <.05 were considered as statistically significant. PIRA: progression independent of relapse activity, PIRA⁻: no evidence of PIRA, RRMS: relapsing remitting multiple sclerosis, MRI: magnetic resonance imaging, TIV: total intracranial volume, BP: whole brain parenchymal volume, THAL: thalamus volume, HIPP: hippocampal volume, BVL: brain volume loss, FSMC-M: Fatigue Scale for Motor and Cognitive Functions (motoric), FSMC-C: Fatigue Scale for Motor and Cognitive Functions (cognitive).

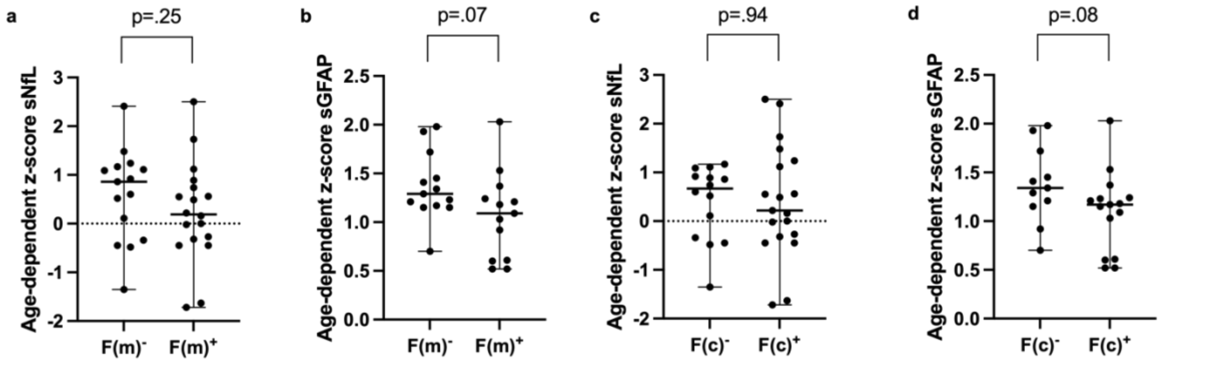


**Figure S2 Group comparisons of clinically stable RRMS (PIRA⁻) patients with (F(m)⁺/F(c)⁺) and without (F(m)⁻/F(c)⁻) motoric (F(m)) or cognitive (F(c)) fatigue regarding sNfL and sGFAP**

We performed group comparisons using Mann-Whitney-U test between clinically stable RRMS (PIRA⁻) patients with (F(m)⁺, n=30; F(c)⁺, n=31) and without motoric or cognitive fatigue (F(m)⁻, n=24; F(c)⁻, n=23) according to the FSMC-M (**a; b**) and FSMC-C (**c; d**). (**a; c**) age-dependent z-score for sNfL and (**b; d**) age-dependent z-score for sGFAP. Values are displayed with median and range. P-values <.05 were considered as statistically significant. PIRA: progression independent of relapse activity, PIRA⁻: no evidence of PIRA, RRMS: relapsing multiple sclerosis, sNfL: serum neurofilament light chain, sGFAP: serum glial fibrillary acidic protein, FSMC-M: Fatigue Scale for Motor and Cognitive Functions (motoric), FSMC-C: Fatigue Scale for Motor and Cognitive Functions (cognitive).

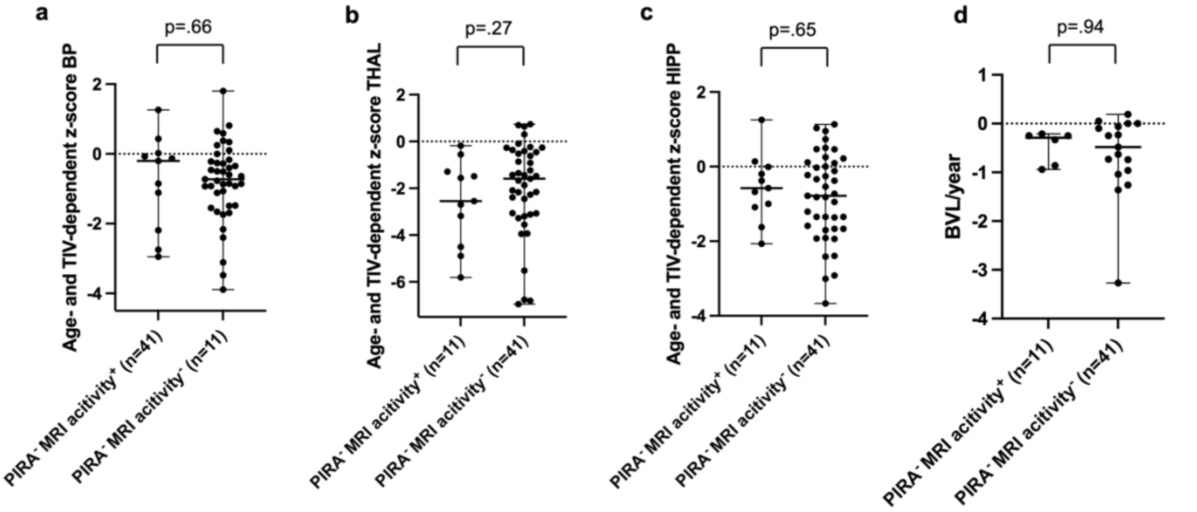

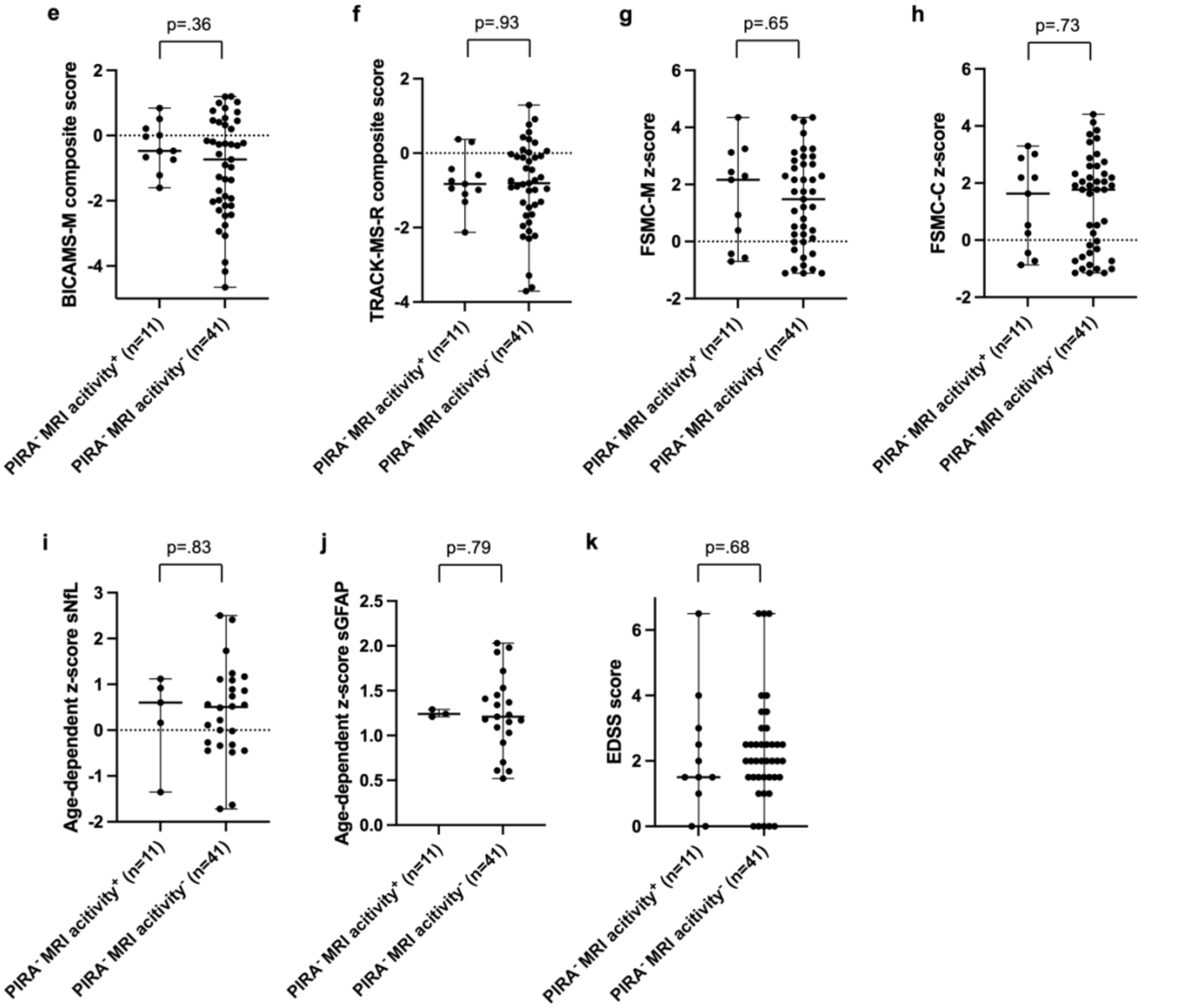


**Figure S3 Group comparisons of 52 clinically stable RRMS (PIRA$^-$) patients with and without MRI activity.**

We performed group comparisons using Mann-Whitney-U test between clinically stable RRMS (PIRA$^-$) patients with (n=11) and without MRI activity (n=41) in a follow-up screening (*M*=1.001 years, range (.28-3.82). (**a**) age- and TIV-dependent z-score for BP, (**b**) age- and TIV-dependent z-score for THAL, (**c**) age- and TIV-dependent z-score for HIPP, (**d**) BVL/year, (**e**) BICAMS-M composite score, (**f**) TRACK-MS-R composite score, (**g**) FSMC-M z-score, (**h**) FSMC-C z-score, (**i**) age-dependent z-score sNfL, (**j**) age-dependent z-score sGFAP, and (**k**) EDSS score. Values are displayed with median and range. P-values <.05 were considered as statistically significant. PIRA: progression independent of relapse activity, PIRA$^-$: no evidence of PIRA, RRMS: relapsing multiple sclerosis, MRI: magnetic resonance imaging, M: median, TIV: total intracranial volume, BP: whole brain parenchymal volume, THAL: thalamus volume, HIPP: hippocampal volume, BVL: brain volume loss, BICAMS-M: German version of the “Brief International Cognitive Assessment for Multiple Sclerosis”, FSMC-M: Fatigue Scale for Motor and Cognitive Functions (motoric), FSMC-C: Fatigue Scale for Motor and Cognitive Functions (cognitive), sNfL: serum neurofilament light chain, sGFAP: serum glial fibrillary acidic protein, EDSS: Expanded disability status scale.
